# Biodegradation of Bisphenol A, Bisphenol F and Bisphenol S in Seawater

**DOI:** 10.3390/ijerph6041472

**Published:** 2009-04-17

**Authors:** Erica Danzl, Kazunari Sei, Satoshi Soda, Michihiko Ike, Masanori Fujita

**Affiliations:** Division of Sustainable Energy and Environmental Engineering, Graduate School of Engineering, Osaka University, 2-1 Yamada-oka, Suita, Osaka 565-0871, Japan; E-mails: sei@see.eng.osaka-u.ac.jp (K.S.); soda@see.eng.osaka-u.ac.jp (S.S.); ike@see.eng.osaka-u.ac.jp (M.I.); mf@env.eng.osaka-u.ac.jp (M.F.)

**Keywords:** Biodegradation, bisphenol A (BPA), bisphenol (BPF), bisphenol S (BPS), TOC Handai, sea die-away

## Abstract

A group of compounds structurally similar to bis(4-hydroxyphenyl)propane (bisphenol A, BPA) are called bisphenols (BPs), and some of them can partially replace BPA in industrial applications. The production and consumption of BPs other than BPA, especially those of bis(4-hydroxyphenyl)methane (bisphenol F, BPF) and bis(4-hydroxyphenyl)sulfone (bisphenol S, BPS), have increased recently, leading to their detection as contaminants in the aquatic environment. The three compounds tested 100% positive for estrus response in 1936 and concerns about their health risks have been increasing. Abundant data on degradation of bisphenols (BPs) has been published, but results for biodegradation of BPs in seawater are lacking. However, several research groups have focused on this topic recently. In this study, the biodegradation behaviors of three BPs, namely BPA, BPF and BPS, in seawater were investigated using TOC Handai (TOC, potential test) and river (sea) die-away (SDA, simulation test) methods, which are both a kind of river-die-away test. The main difference between the tests is that indigenous microcosms remain in the sampled raw seawater for the SDA experiments, but they are removed through filtration and dispersed into artificial seawater for the TOC experiments. The BPs, except for BPS, were degraded using both methods. The SDA method produced better biodegradation results than the TOC method in terms of degradation time (both lag and degradation periods). Biodegradation efficiencies were measured at 75–100% using the SDA method and 13–63% using the TOC method. BPF showed better degradation efficiency than BPA, BPF was > 92% and BPA 83% depleted according to the SDA tests. BPS degradation was not observed. As a conclusion, the biodegradability of the three BPs in seawater could be ranked as BPF > BPA ≫ BPS. BPF is more biodegradable than BPA in seawater and BPS is more likely to accumulate in the aquatic environment. BPS poses a lower risk to human health and to the environment than BPA or BPF but it is not amenable to biodegradation and might be persistent and become an ecological burden. Thus other degradation methods need to be found for the removal of BPS in the environment.

## 1. Introduction

**Figure 1.**
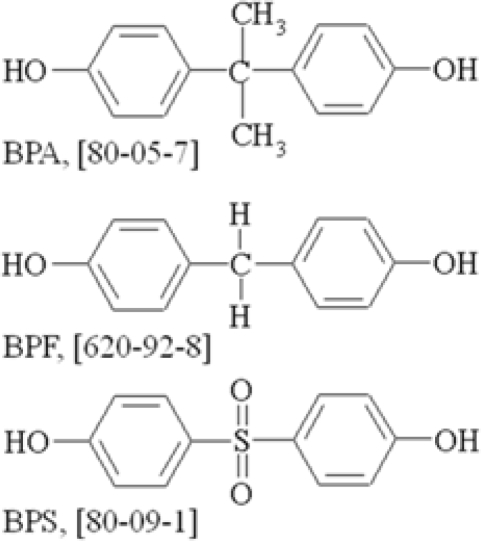
Formulas and CAS numbers in brackets for bisphenol A (BPA), bisphenol F (BPF) and bisphenol S (BPS).

BPA, BPF and BPS (Figure 1) are monomers used in the resin and plastic industry to produce lacquers, the inner coating of food cans and thermal paper [1–3]. Concerns about their health risks are increasing. In 2005 the European Union banned the manufacture of bisphenol F diglycidyl ether as a food packaging material [4]. Dodds and Lawson [5] stated that diphenyl and diphenyl methane derivates containing two hydroxyl groups in the *para* positions, amongst them BPA, BPF and BPS, showed estrogenic activity. The three compounds mentioned tested 100% positive for estrus response. Abundant data on estrogenic activity, androgen activity, carcinogenicity and toxicity has been published since then, mostly on BPA, and recently low-dose effects of BPA have been discussed [6,7]. Estrogenic activity of BPA was similar to that of BPF, whereas BPS showed weak activity in a yeast two-hybrid assay [8]. BPA exhibited higher toxicity than BPF or BPS on *Daphnia magna* and the 48-h EC_50_ values for BPA, BPF and BPS were 10 mg/L, 56 mg/L and 55 mg/L, respectively.

The production and consumption of BPs and therefore their discharge into the aquatic environment, has tended to increase. BPs have been detected in the aquatic environment and reported in literature [9–13]. Thus the fate of these compounds in the aquatic environment is of great concern in terms of both human health and environmental risks.

Reports on biodegradation of BPs in seawater are scarce. Kang and Kondo [14] confirmed rapid biodegradation of BPA under aerobic conditions in river water. In seawater however, a lag period of 30–40 days was observed and BPA was degraded within 60 days. Changes in bacterial counts did not correlate with BPA degradation and the authors suggested chemical degradation to be responsible for depletion of the compound in seawater. An immediate BPA-degradation-onset and a half-life of 14.5 days in marine sediment under aerobic conditions was reported by Ying and Kookana [15]. They observed a lag time of 35 days in seawater and that the chemical was almost degraded on day 56 under aerobic conditions. Sakai *et al.* [16] isolated from seawater the bacterial strain *Spingomonas* sp. strain BP-7, which was able to degrade BPA and some other related compounds. The strain could not degrade BPS, and BPF inhibited its growth.

The aim of this study is to investigate biodegradation of BPA, BPF and BPS in seawater applying the TOC and SDA methods. The TOC method uses the seawater microorganisms collected through filtration from natural (raw) seawater at two sites in the bay of Osaka. The retained organisms are then dispersed into artificial seawater at original levels of cell density. The SDA method uses samples with the indigenous seawater microorganisms in their natural environment – the natural (raw) seawater sampled from two sites in the bay of Osaka.

Our laboratory has been using the TOC method mainly for experiments with river water and has published the results [17]. Preliminary studies have also been performed using the TOC method as applied to seawater and the results have been reported in a diploma thesis (data not published). One of the main questions for the current study is: Is the TOC method applicable to seawater and do the results differ from those obtained using the SDA method? The TOC method was the method of choice in the laboratory but we needed to compare the experiments using a method in which the microorganisms were less influenced by conditions in the laboratory. For the TOC tests, microorganisms are supplied with nutrients necessary in a model environment and they acclimate to the artificial environment. For the SDA tests the microorganisms remain in their natural habitat and thus might have an advantage in biodegradation.

## 2. Materials and Methods

### 2.1. Chemicals and Reagents

Artificial Seawater (ASW) (Yashima Pure Chemicals, Osaka, Japan) was prepared in ultra pure water (UPW, Direct-Q, Millipore, Molsheim, France) according to the producer’s instructions and fortified with KNO_3_ and NaH_2_PO_4_·2H_2_O as nitrogen and phosphorus sources. Stock solutions of 4,4′-isopropylidenediphenol (BPA, bisphenol A, C_15_H_16_O_2_), 4,4′-methylenediphenol (BPF, bisphenol F, C_13_H_12_O_2_) and 4,4′-sulfonyldiphenol (BPS, bisphenol S, O_2_S(C_6_H_4_OH)_2_) (all three from TCI chemicals, Tokyo, Japan) were prepared in UPW, heated to dissolve and filter-sterilized (0.20 μm, Dismic-25cs, Advantec, Toyo Roshi, Japan). The total organic carbon (TOC) concentrations of the solutions were measured with a Shimadzu TOC-5000 A instrument.

Metabolite stock solutions of 1,4-benzoquinone (C_6_H_4_O_2_), 1,4-hydroquinone (C_6_H_4_(OH)_2_), 4-hydroxybenzoic acid (C_7_H_6_O_3_), (all three Kishida chemicals, Osaka, Japan) and 4,4’-dihydroxybenzophenone ((HOC_6_H_4_)_2_CO) (TCI chemicals, Tokyo, Japan) were prepared in UPW.

### 2.2. Samples

Seawater was collected at two sites in the bay of Osaka, Japan: one located next to the Asia and Pacific Trade Center in the South port area of the city (+34° 38′ 12.39″, +135° 24′ 41.67″) and the other one close to the Osaka Hokko lime wharf in the North port area (+34° 39′ 53.30″, +135° 25′ 15.35″). Seawater was collected from 30–50 cm below the surface. Sufficient headspace was provided to ensure aerobic conditions of the bottled water and the samples were kept in a cooling box supplied with ice packs at 4°C. To roughly purify the seawater it was filtered through 10.0 μm filters (Omnipore Membrane Filters, Millipore, Ireland) upon arrival at the laboratory and processed within approximately 12 hours after sampling.

### 2.3. Heterotrophic Bacterial Counts

Heterotrophic bacteria were measured by plate counts on 1/10 CGY media containing casitone 0.5 g, glycerin 0.5 g, yeast 0.1 g and agar 15 g dissolved in 1 L of ASW. Serial dilutions of 10.0 μm filtered seawater were prepared by diluting a 0.05% sodium tripolyphosphate solution with ASW. Triplicate petri dishes were incubated at 28°C for 7–10 days after spreading 100 μL-aliquots of the dilutions onto the media (plates). The water quality parameters and colony forming units (CFU) are listed in Table 1.

### 2.4. Biodegradation Tests of BPs

Biodegradation of BPA, BPF and BPS was tested using modified TOC Handai [18] and modified river (sea) die-away [19] methods conducted in parallel. Samples, except for the experiment blanks were amended with BPA, BPF or BPS as carbon source with TOC concentrations of 4–9 mg/L. The total sample volume was 50 mL in 70 mL test tubes. Degradation tests were performed in duplicate and all samples were incubated at 28°C with shaking at 120 rpm in the dark for 30–60 days. Aliquots of 2 mL were withdrawn periodically and centrifuged at 18–600 × G or 16–060 × G for 20 or 30 min. The supernatants were kept at −20°C for further analysis.

*Modified TOC Handai method* Ten times concentrated microorganism solutions were generated by filtering 500 mL of the purified seawater with 0.22 μm filters (Durapore Membrane Filters, Millipore, Ireland) followed by 10 minutes sonication (130 W) of the filters to disperse the filter-collected microorganisms into sterilized ASW. Concentrated sterile ASW was diluted into sterilized UPW, inoculated with 5 mL of concentrated microorganism solution and supplemented with one of the three test chemicals. The pH was adjusted to 7.4 ± 0.3. Blank samples were not fortified with any test chemical and were prepared for each sampling point. Three non-inoculated controls were prepared as well.

*River die-away/Sea die-away method* Aliquots of the purified seawater were added to the test chemicals in sterilized test tubes. Blanks that did not contain any bisphenols were prepared for each sampling point. The water for the control samples was autoclaved at 121°C for 20 min before aliquoting into the appropriate tubes. Separate controls were prepared for the three test chemicals and for each sampling point giving 6 controls in total. The pH was adjusted to the value of the blank sample, which was usually only necessary for the control samples.

### 2.5. Analysis of BPA, BPF and BPS

Concentrations of the test chemicals were determined by eluting the samples with an acetonitrile (HPLC grade, Cica Reagent Kanto Chemicals, Tokyo, Japan) to water ratio of 1:1 (v/v) and isocratic flow of 1 mL/min from a reversed-phase column (GVP-ODS guard column, 10 × 4.6 mm I.D.; VP-ODS packed column, 150 × 4.6 mm I.D., both Shim-Pack, Shimadzu) attached to a Shimadzu HPLC system equipped with a SCL-10A VP system controller, DGU-14A degasser, two LC-10AD VP pumps and a SIL-10AF auto injector device. The CTO-10A VP column oven temperature was set to 40 °C and the component peaks were detected at a wavelength of 280 nm on a SPD-10A VP UV-VIS detector. The retention times (t_R_) of the bisphenols were: t_R BPA_ = 4.5 min, t_R BPF_ = 3.5 min and t_R BPS_ = 2.5 min.

## 3. Results

**Figure 2.**
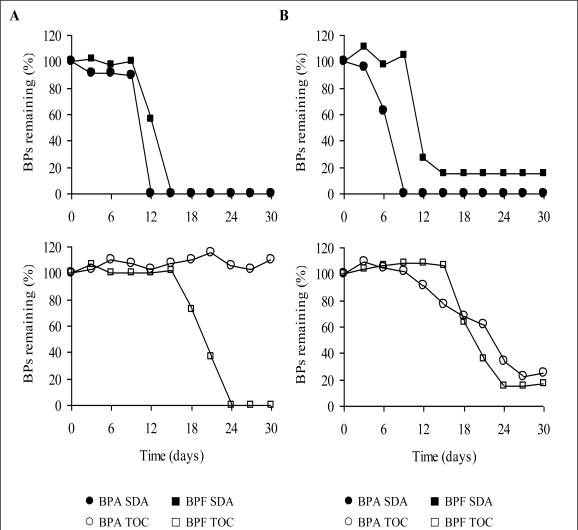
Typical time courses for bisphenol A and F (BPs) biodegradation in January (A) and in October (B) for South port samples. Top: Sea die-away method (SDA). Bottom: TOC Handai method (TOC).

Typical time courses for BPA and BPF degradation measured using the TOC and SDA methods in January and October are shown in Figure 2. Faster degradation followed shorter lag periods, as measured using the SDA method. The lag periods for all experiments degrading BPA using the SDA method were between 0–15 days and the microcosms exhibited degradation within 3–12 days. 6–12 days were needed for degradation following a lag period of 3–21 days for all experiments degrading BPF using the SDA method.

The lag periods for the 2 samples degrading BPA using the TOC method were between 6 and 12 days, and the degradation times between 12 and 21 days, whereas microcosms exhibited long lag periods (12–45 days) as well as long degradation times (9–39 and more days) for BPF degradation. The BPs’ concentrations in the control samples of both methods did not change noticeably. Furthermore, the BPS concentration remained unchanged over the test period of 60 days in the TOC and SDA samples. BPA was hardly degraded using the TOC method. No test (out of six) showed BPA degradation in the North port samples while two out of six of the South port samples (33% degradation efficiency) could degrade BPA using the TOC method. Tables 2 and 3 represent degradation efficiencies of BPA, BPF and metabolites. Degradation tests for the North port samples showed no BPF reduction (four samples) in December and October experiments and partial and complete degradation for the two samples in the January experiments, representing 17% BPF degradation efficiency in the North port tests as measured by the TOC method. BPF showed 83% degradation efficiency and was degraded in five out of six TOC samples, out of which two showed BPF depletion within 30 days in the South port tests. In general BPF could be degraded in all but one sample in the South port experiments and according to both methods within 48 days. All the SDA samples degraded BPA (six samples) and BPF (seven samples) within 30 days in the South port tests. Degradation efficiencies were similar for BPA and BPF and both sampling points as measured by the SDA method, except for the January experiments in which no depletion of BPA (two samples) could be detected in the North port tests.

Thus four tests out of six depleted BPA (67% degradation efficiency) in the North port sampled water using the SDA method. The North port samples exhibited better BPF degradation using the SDA method than using the TOC method and 83% BPF degradation efficiency were measured by the SDA method. According to the SDA method four samples (out of six) degraded BPF completely in the North port microcosms during a 30-days-period. One of two samples that showed little and partial degradation was degraded on day 42, but for the other sample further data is missing because monitoring was stopped on day 30. Summarizing the results for BPA and BPF for both sampling sites and using both methods we find an overall biodegradation efficiency of 8% in the North port and 58% in the South port tests as measured by the TOC method and 75% in the North port and 100% in the South port tests as measured by the SDA method.

Because of the difference in water temperatures (Table 1) experiments can be split into two experimental seasons for the data interpretation in this study: ‘Winter’ for January and December and ‘Summer’ with high water temperatures for October. Comparison of summer and winter experimental data shows that BPF was degraded in approximately half of the microcosms according to the TOC method (four completely plus one incompletely degraded sample out of eight in winter compared with two completely degraded samples out of four in summer) during both seasons. Thus degradation efficiencies were 50% in winter as well as in summer for BPF as measured by the TOC method. However, little degradation of BPA was observed in winter (13% efficiency) as well as in summer (25% efficiency) tests using the TOC method. Using the SDA method hardly any seasonal degradation differences of BPF were measured and its overall biodegradation efficiency was 92% due to one sample that was withdrawn on day 30. Data showed that the sample was likely to have degraded in less than one more week, so biodegradation efficiencies for BPF should be 100% for summer as well as for winter. BPA degradation in winter (75% efficiency) was less efficient than in summer with 100% degradation efficiency: two of the winter microcosms (four) did not degrade the compound according to the SDA method.

Metabolites were detected during BPA degradation and the TOC samples had more metabolites than the SDA samples in December and October (Table 4). Winter experiments had a peak at 2.4 min in common and a peak at 2.6 min was detected for the TOC experiments in December as well as for the SDA experiments in January. The t_R_ differed between summer and winter experiments. However, all the SDA samples’ metabolites had the same t_R_ as the TOC samples’ metabolites in October, excluding the latest at 3.6 min (TOC) and 4.6 min (SDA) and adding two for the TOC method.

The metabolites with t_R_ between 2.2–2.7 min in the TOC December experiments were not degraded within 60 days, whereas the metabolites with t_R_ of 2.0, 2.5, 3.4 and 4.6 min were found to be persistent in the October experiments according to the SDA method.

Generally, October experiments showed more metabolite peaks than all other experiments for BPA as well as for BPF (Table 5). According to the SDA method more metabolite peaks occurred during BPF degradation than the TOC method in October. The TOC and the SDA results showed metabolite peaks in common at 2.0 and 2.2 min in October. Ultimately, the only metabolite that persisted had a t_R_ of 2.3 min in the TOC experiment in January. The sample showed poor BPF degradation as well.

Metabolite peaks occurring during BPA and BPF degradation overlapped with the peaks of benzoquinone, hydroquinone or hydroxybenzoic acid but could not be clearly identified as such through chromatogram comparison or spiking. The metabolite peak detected at t_R_ = 2.6 min occurring during BPF degradation could be identified as dihydroxybenzophenone through chromatogram comparison and spiking of the sample with dihydroxybenzophenone.

**Figure 3.**
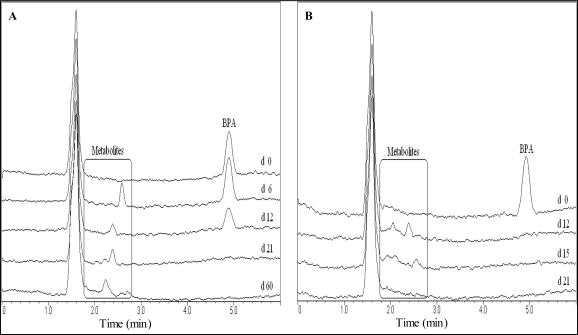
HPLC chromatogram showing BPA degradation and metabolites in seawater with partial degradation **(A)** and complete degradation **(B)**. Day (d) of sampling is given.

**Figure 4.**
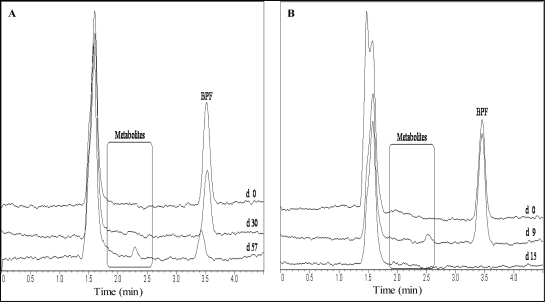
HPLC chromatogram showing BPF degradation with metabolites in seawater with partial degradation **(A)** and complete degradation **(B)**. Day (d) of sampling is given.

Analytical results for partial and complete degradation of the BPs are compared in Figures 3 and 4. The time-frame of occurring metabolites is depicted. In samples with complete degradation of BPs as well as metabolites, no corresponding peaks were ultimately detected.

Results in other experiments revealed BPA and BPF metabolite peaks occurring at t_R_ later than the HPLC-program run times of 6.0 (BPA) and 4.5 (BPF) min. The run times were extended in the October experiments and peaks at t_R_ = 10.0 (BPA experiments) and t_R_ = 4.6 min (BPF experiments) were recorded regularly. These peaks were depleted at the same time or before the parent compounds. The observed peaks remained unchanged in samples showing no degradation, including the control samples. In one TOC and two SDA samples metabolites with t_R_ of 4.6, 5.6 and 5.9 min were detected that depleted within 30 days.

Summarizing the results in Table 6, we see that BPA and BPF were degraded more easily using the SDA method. All samples degraded the compound added in the SDA experiments except for the winter samples, in which half of the BPA tested microcosms did not exhibit any degradation, and except for the BPF sample for which the experiment was terminated on day 30. Biodegradation efficiencies were more than 92% for BPF and 83% for BPA in the SDA experiments. Using the TOC method a difference in BPA and BPF degradation ability of the microcosms is apparent. BPA degradation occurred in two out of 12 samples (17% degradation efficiency) whereas BPF degradation occurred in six out of 12 samples (50% efficiency).

## 4. Discussion

Degradation of BPS could not be detected in this study, a finding observed in river water too [17]. Therefore, BPS might accumulate and remain in the environment for a long time. Sakai *et al.* [16] found that during a 28 day test period in seawater, 18% of BPA was biodegraded whereas 67% of BPA was biodegraded in activated sludge that had been diluted into salt solution. The authors [16] described seawater as a nutrient poor environment. Ying and Kookana [15] who detected greater than 90% BPA degradation both in seawater and in marine sediment referred to differences such as a more active and diverse microbial community, as well as a richer nutrient environment in the sediment samples that might explain the lack of an acclimation period in the marine sediment. In the seawater, a lag period of 35 days had been observed. Our experiments revealed a possible influence of microbial communities and nutrient supply or composition on biodegradation, too. Some differences in biodegradation might be attributed to the varying number of CFU in this study. Higher counts of CFU in the North port sampled water in December had no influence on BPA or BPF degradation in the TOC experiments. In both cases only South port water samples showed degradation of the compounds to some extent. The SDA samples were not affected by changes in CFU in December and October experiments. However, the 9x higher CFU counts in January in the South port water showed some effect: BPA degradation was observed only in the South port tests and not in the North port ones, whereas degradation of BPF was complete within 30 days in all South port tests and one (out of two) North port test. The remaining sample took 42 days to degrade the compound. The CFU in January in the North port water were the lowest among the sampling points and months.

The TOC method might be more sensitive to limitation of or excess in nutrients and the fact that the microcosms are extracted from environmental samples to be dispersed into artificial sea water should be kept in mind. The method is designed to supply microorganisms with optimum nutrient conditions except for the carbon source. For the seawater experiments however, the method had to be modified. Fortification of the commercially available ASW, of which detailed information about its composition is unavailable, with nitrogen and phosphate might be insufficient and cause the lower biodegradation efficiencies observed. Furthermore, the availability of nitrogen that was supplied as KNO_3_ could have had an impact on the results, and fortification with NH_4_ might have improved method performance. Moreover, the pH using the TOC method was adjusted to 7.4 ± 0.3. However, the only sample showing this pH value in the natural environment was the North port sample in January.

Kang and Kondo [14] hypothesized that BPA was degraded chemically by reactive oxygen species present in seawater and that bacteria and flagellates might have an important effect on the degradation process. The group placed autoclaved and non-autoclaved seawater samples at 4, 25 and 35°C for 60 days, supplied oxygen through air bubbling and observed lag periods between 30 and 45 days and BPA degradation between 40 and 60 days even though bacterial counts were low. Thus BPA degradation could not be related to the change in bacterial counts, which had increased during the first 3–5 days of the test period and decreased afterwards. The control samples did not show any change in BPA concentration, a finding that was confirmed by the present study. In the current study, autoclaved and non-autoclaved samples were prepared too, but placed in the dark at 28°C in a rotary shaker to provide aeration. Degradation of BPA and BPF within 30 days was found and the samples were assigned completely degraded, whereas samples that degraded the chemicals within 60 days were assigned partially (intermediate) degraded, dependent on the remaining concentrations of the chemicals or on remaining metabolites. Dorn *et al.* [20] as well as Klečka *et al.* [19] stated that there were no changes or negligible losses in the concentration of the controls confirming that biodegradation occurred in the biodegradation tests. We summarize the facts indicating that microbial degradation is the cause for depletion of BPA and BPF in the non-autoclaved seawater samples in the present study: The fact that the compound concentrations in the control samples did not noticeably change over a 60 days period and the fact that samples were incubated in the dark thus chemical degradation was obviated.

## 5. Conclusions

Neither BPA nor BPF were degraded in the control samples, which consisted of microcosms-removed (TOC) or autoclaved (SDA) test or sea water, implying that the microcosms-including biodegradation samples degraded the compounds. This report is the first about BPF-biodegradation in seawater and supports the findings of Sakai *et al.* [16] and Ying and Kookana [15] that BPA is biodegraded in seawater.

Differences in BPA and BPF degradation might also result from the chemical structures. The hydrogen atoms (BPF) attached to the quaternary carbon might be an easier site of attack for the microorganisms than the methyl groups attached to that central atom in BPA.

This study is a first approach to describe metabolites occurring during degradation of BPs in seawater. Out of 18 samples with metabolite detection, metabolites in three BPA samples (one TOC and two SDA) and one BPF sample (TOC) were found persistent after 60 days. Metabolites shall be further analyzed and identified through e.g. chromatogram comparison with known compounds and spiking as well as MS analysis.

The results clearly show that BPA and BPF are biodegradable in seawater. 88% of the compounds were degraded using the SDA method and 33% degraded using the TOC method. Therefore we suggest using the SDA method for biodegradation experiments.

## Figures and Tables

**Table 1.** Water quality parameters and colony forming units (CFU) for the North and South port sampling sites.

	December 2005		January 2006		October 2006	
	North	South	North	South	North	South
pH	8.1	8.0	7.4	8.2	7.9	7.9
Turb. (mg/L)	12	11	8	14	0	1
DO (mg/L)	10.7	8.9	11.4	9.0	4.3	4.8
Temp. (°C)	8.6	9.8	8.4	8.4	23.2	23.4
Cond. (S/m)	4.5	4.3	4.9	4.6	4.6	4.1
CFU/mL	5.3 × 10^3^	2.4 × 10^3^	3 × 10^2^	2.6 × 10^3^	3.8 × 10^2^	7.5 × 10^2^

Turb. Turbidity

Cond. Conductivity

**Table 2.** Degradation of bisphenol A (BD) and its metabolites (MD) as measured by the TOC Handai (TOC) and the Sea die-away (SDA) methods and monitored by HPLC.

	December 2005				January 2006^a^			October 2006			
	TOC		SDA		TOC	SDA		TOC		SDA	
	BD	MD	BD	MD	BD	BD	MD	BD	MD	BD	MD
N1	−	nd	+++	+++	−	−	nd	−	nd	+++	+++
N2	−	nd	+++	+++	−	−	nd	−	nd	+++	+++
S1	−	nd	+++	nd	−	+++	−(42)	++(33)	++(36)	+++	++
S2	+++	−	+++	nd	−	+++	+++	−	nd	+++	++

Results for a 30 days period and day of degradation, if occurred within 60 days after sampling, as numbers in brackets.

+++ complete degradation (> 90%)

++ degradation (50 to 90%)

+ little degradation (10 to 50%)

− no degradation (< 10%)

nd not detected

N North port samples 1, 2

S South port samples 1, 2

^a^ No metabolites detected in TOC method

**Table 3.** Degradation of bisphenol F (BD) and its metabolites (MD) as measured by the TOC Handai (TOC) and the Sea die-away (SDA) methods and monitored by HPLC.

	December 2005^a^			January 2006				October 2006			
	TOC	SDA		TOC		SDA		TOC		SDA	
	BD	BD	MD	BD	MD	BD	MD	BD	MD	BD	MD
N1	−	+++	+++	+++	nd	+(42)	nd	−	nd	++^b^	nd
N2	−	+++	nd	+	−	+++	nd	−	nd	+++	nd
S1	+++	+++	nd	++(48)	nd	+++	nd	++(36)	+++	+++	+++
S2	−	+++	nd	+++	nd	+++	+++	++(36)	+++	+++	+++
S3						+++	+++				

Results for a 30 days period and day of degradation, if occurred within 60 days after sampling, as numbers in brackets.

+++ complete degradation (> 90%)

++ degradation (50 to 90%)

+ little degradation (10 to 50%)

− no degradation (< 10%)

nd not detected

N North port samples 1, 2

S South port samples 1, 2, 3

^a^ No metabolites detected in TOC method

^b^ Experiment stopped on day 30

**Table 4.** BPA metabolite list for a 60 days test period.

	TOC		SDA		
	Dec	Oct	Dec	Jan	Oct
t_R_ (min)					
BPA	4.9	4.5	4.9	4.9	4.5
	2.0	1.9	2.4	1.9	1.9
	2.2	2.0		2.1	2.0
	2.4	2.2		2.4	2.2
	2.6	2.3		2.6	2.4
	2.7	2.4			2.5
		2.5			2.6
		2.6			3.4
		3.1			4.6
		3.4			
		3.6			

**Table 5.** BPF metabolite list for a 60 days test period.

	TOC		SDA		
	Jan	Oct	Dec	Jan	Oct
t_R_ (min)					
BPF	3.5	3.4	3.5	3.5	3.4
	2.3	2.0	2.5	2.2	2.0
		2.2			2.2
					2.6
					3.1

**Table 6.** Summary of results for a 60 day monitoring period.

	TOC						SDA					
	Winter (8)			Summer (4)			Winter BPA (8) BPF (9)			Summer (4)		
	C	I	N	C	I	N	C	I	N	C	I	N
BPA	1	0	7	1	0	3	6	0	2	4	0	0
BPF	4	1	3	2	0	2	9	0	0	3	1^a^	0

Data represent number of microcosms exhibiting complete (C), intermediate (I) and no (N) BPs degradation as monitored by HPLC. Numbers in brackets show the total number of microcosms.

^a^ Experiment stopped on day 30
